# Update of the Blood Lead Reference Value — United States, 2021

**DOI:** 10.15585/mmwr.mm7043a4

**Published:** 2021-10-29

**Authors:** Perri Zeitz Ruckart, Robert L. Jones, Joseph G. Courtney, Tanya Telfair LeBlanc, Wilma Jackson, Mateusz P. Karwowski, Po-Yung Cheng, Paul Allwood, Erik R. Svendsen, Patrick N. Breysse

**Affiliations:** 1 National Center for Environmental Health/Agency for Toxic Substances and Disease Registry, CDC.

The negative impact of lead exposure on young children and those who become pregnant is well documented but is not well known by those at highest risk from this hazard. Scientific evidence suggests that there is no known safe blood lead level (BLL), because even small amounts of lead can be harmful to a child’s developing brain (*1*). In 2012, CDC introduced the population-based blood lead reference value (BLRV) to identify children exposed to more lead than most other children in the United States. The BLRV should be used as a guide to 1) help determine whether medical or environmental follow-up actions should be initiated for an individual child and 2) prioritize communities with the most need for primary prevention of exposure and evaluate the effectiveness of prevention efforts. The BLRV is based on the 97.5th percentile of the blood lead distribution in U.S. children aged 1–5 years from National Health and Nutrition Examination Survey (NHANES) data. NHANES is a complex, multistage survey designed to provide a nationally representative assessment of health and nutritional status of the noninstitutionalized civilian adult and child populations in the United States (*2*). The initial BLRV of 5 *μ*g/dL, established in 2012, was based on data from the 2007–2008 and 2009–2010 NHANES cycles. Consistent with recommendations from a former advisory committee, this report updates CDC’s BLRV in children to 3.5 *μ*g/dL using NHANES data derived from the 2015–2016 and 2017–2018 cycles and provides helpful information to support adoption by state and local health departments, health care providers (HCPs), clinical laboratories, and others and serves as an opportunity to advance health equity and environmental justice related to preventable lead exposure. CDC recommends that public health and clinical professionals focus screening efforts on populations at high risk based on age of housing and sociodemographic risk factors. Public health and clinical professionals should collaborate to develop screening plans responsive to local conditions using local data. In the absence of such plans, universal BLL testing is recommended. In addition, jurisdictions should follow the Centers for Medicare & Medicaid Services requirement that all Medicaid-enrolled children be tested at ages 12 and 24 months or at age 24**–**72 months if they have not previously been screened (*3*).

## Methods, Rationale, and Evidence

CDC has been involved in defining the criteria for interpreting BLLs in children since 1971 (*4*) (Table 1). The criteria for interpreting BLLs in children was revised over time based on new clinical and scientific evidence and improved laboratory technologies.

**TABLE 1:** Definitions for interpreting children's blood lead levels — United States, 1960–2021

Year	Blood lead level (*μ*g/dL)	Interpretation*
1960	60	NA
1970	40	Undue or increased lead absorption
1975	30	Undue or increased lead absorption
1978	30	Elevated blood lead level
1985	25	Elevated blood lead level
1991	10	Level of concern
2012	5	Reference value
2021	3.5	Reference value

**Abbreviation:** NA = not available.

* https://stacks.cdc.gov/view/cdc/61820

In 2012, CDC’s former Advisory Committee on Childhood Lead Poisoning Prevention (ACCLPP) recommended the establishment of the BLRV and proposed it be set at 5 *μ*g/dL (*5*). This recommendation was based on the weight of evidence indicating that the adverse health effects of BLLs <10 *μ*g/dL in children included neurologic, cardiovascular, immunologic, and endocrine effects. ACCLPP further recommended that the BLRV be updated every 4 years based on the 97.5th percentile of BLLs for children aged 1–5 years across the two most recent combined NHANES cycles for which data are available.

The Lead Exposure and Prevention Advisory Committee (LEPAC) was established under the Water Infrastructure Improvements for the Nation Act of 2016.* The LEPAC is charged with providing advice and guidance to the Secretary of U.S. Department of Health and Human Services (HHS), Director of CDC, and Administrator of Agency for Toxic Substances and Disease Registry on matters related to lead poisoning prevention and surveillance. In 2020, LEPAC charged a BLRV workgroup with providing advice and guidance regarding new scientific knowledge and technological developments to guide the BLRV. During a May 2021 meeting of the LEPAC, the workgroup recommended that the BLRV be updated from 5 *μ*g/dL to 3.5 *μ*g/dL using data derived from the two most recent NHANES cycles (2015–2016 and 2017–2018), and the LEPAC voted unanimously to accept this recommendation (*6*). Subsequently, the committee submitted a formal recommendation to the HHS Secretary to update the BLRV from 5 *μ*g/dL to 3.5 *μ*g/dL. LEPAC’s BLRV workgroup also advised that CDC address barriers and capacity issues for federal agencies and other partners to facilitate adopting the revised BLRV (*6*). This will include providing training and outreach to public health professionals. The HHS Secretary and CDC concur with the recommendation and have developed communication and implementation plans to announce and promote the BLRV update, including to those at greatest risk.

## Policy Update

The BLRV is a population-based measurement which indicates that 2.5% of U.S. children aged 1–5 years have BLLs ≥3.5 *μ*g/dL. It is not a health-based standard or a toxicity threshold. The BLRV should be used as a guide to 1) help determine whether medical or environmental follow-up actions should be initiated for an individual child and 2) prioritize communities with the most need for primary prevention of exposure and evaluate the effectiveness of prevention efforts. Whether a BLL measurement at or above the BLRV triggers medical or environmental follow-up will depend on existing jurisdictional laws, regulations, and resource availability. Follow-up lead testing to confirm BLLs is recommended. CDC strongly advises that providers follow CDC’s Recommended Actions Based on Blood Lead Level (*7*).

## Discussion

The geometric mean BLL in U.S. children aged 1–5 years has declined over time from 15.2 *μ*g/dL in 1976–1980 to 0.83 *μ*g/dL in 2011–2016 (Table 2) (*8*). During the same period among U.S. children aged 6–11 years, the geometric mean BLL declined from 12.7 *μ*g/dL to 0.6 *μ*g/dL. Despite this overall declining trend in geometric mean BLLs in U.S. children aged 1–5 years, certain children remain at substantial risk for exposure to lead and disproportionately experience negative health consequences. Their ongoing lead exposure reflects persistent structural inequities in the built environment and access to health care. In addition, the pernicious and irreversible effects of lead exposures perpetuate ongoing structural inequities and injustices (*9*). Thus, lead exposure can be considered both a result and cause of health inequity and environmental injustice.

**TABLE 2:** Weighted geometric mean blood lead levels* in U.S. children aged 1**–**5 years, by selected sociodemographic characteristics — four National Health and Nutrition Examination Survey cycles, United States, 1999–2016

Characteristic	1999–2002		2003–2006		2007–2010		2011–2016	
	No.	GM, *μ*g/dL (95% CI)	No.	GM, *μ*g/dL (95% CI)	No.	GM, *μ*g/dL (95% CI)	No.	GM, *μ*g/dL (95% CI)
**Overall**	1,621	1.95 (1.79–2.12)	1,879	1.61 (1.52–1.71)	1,653	1.33 (1.26–1.41)	2,321	0.83 (0.78–0.88)
**Age group, yrs**								
1–2	779	2.19 (2.01–2.39)	919	1.81 (1.71–1.92)	793	1.49 (1.39–1.59)	1,024	0.93 (0.86–1.00)
3–5	842	1.82 (1.64–2.01)	960	1.48 (1.38–1.60)	860	1.24 (1.15–1.33)	1,297	0.77 (0.72–0.82)
**Sex**								
Male	851	1.95 (1.77–2.14)	951	1.61 (1.51–1.72)	872	1.34 (1.25–1.43)	1,213	0.86 (0.80–0.92)
Female	770	1.95 (1.77–2.16)	928	1.61 (1.49–1.73)	781	1.32 (1.24–1.41)	1,108	0.79 (0.74–0.85)
**Race/Ethnicity^†^**								
Black, non-Hispanic	439	2.81 (2.56–3.09)	530	2.43 (2.12–2.80)	338	1.77 (1.62–1.93)	608	1.07 (0.97–1.18)
Mexican American	541	1.89 (1.75–2.03)	611	1.57 (1.46–1.69)	490	1.28 (1.17–1.39)	526	0.78 (0.72–0.84)
White, non-Hispanic	454	1.83 (1.60–2.09)	535	1.44 (1.35–1.54)	536	1.26 (1.14–1.39)	563	0.79 (0.71–0.88)
**Income to poverty ratio^§^**								
<1.3	808	2.44 (2.24–2.66)	936	2.01 (1.85–2.18)	864	1.57 (1.48–1.67)	1,149	0.97 (0.90–1.05)
≥1.3	686	1.60 (1.45–1.77)	857	1.39 (1.30–1.49)	676	1.17 (1.08–1.27)	997	0.72 (0.67–0.77)

**Abbreviations:** CI = confidence interval; GM = geometric mean; NHANES = National Health and Nutrition Examination Survey.

* Weighted estimates derived from the observed data for the study population using NHANES-specified sampling weights. The GM blood lead levels in children aged 1–5 years have decreased over time.

**^†^** Data by race and Hispanic origin were limited to the three racial and Hispanic origin groups available across all survey cycles (non-Hispanic White, non-Hispanic Black, and Mexican American).

^§^ Computed as the total family income divided by the poverty threshold.

The most common sources of lead exposure in the United States are lead-based paint and dust, lead-contaminated soil, and lead in water from lead pipes and plumbing fixtures (*1*). Other sources of exposure include some toys and jewelry, candies imported from other countries, traditional home remedies, and certain jobs and hobbies that involve working with lead-based products and might cause parents to bring lead into the home. Children who live near airports might be exposed to lead in air and soil from aviation gas. The 2018 Federal Action Plan to Reduce Lead Exposures and Associated Health Impacts^†^ outlines steps that can be taken to reduce lead hazards at the individual, community, and whole system level. In addition, CDC provides extensive guidance on exposure reduction (https://www.cdc.gov/nceh/lead/faqs/lead-faqs.htm). Steps that parents and caregivers can take to reduce lead exposure include becoming more educated about lead hazards; working with a Lead-Safe certified firm^§^ to repair peeling or chipping lead-based paint; replacing lead service lines^¶^; washing children’s hands, bottles, and toys; and removing shoes before entering the home. Lead exposure is not equally distributed across the United States, and young children at highest risk for exposure are those living in housing built before 1978, non-Hispanic Black or African American children, children eligible for Medicaid, and children living in areas with higher poverty rates (*8*). This updated BLRV will drive and support further assessment of BLLs by sociodemographic characteristics which can assist in creating more focused population-based interventions to help address systemic health inequities and environmental injustice. CDC’s Childhood Lead Poisoning Prevention Program (CLPPP) is committed to making new and expanding investments in communities where young children are disproportionately affected by BLLs above the BLRV.

As population BLLs decrease, along with overt clinical signs of lead exposure, laboratory testing has become paramount in detection and subsequent management of lead exposures. Thus, laboratories play an essential role in the overall public health response to lead exposure. A BLRV of 3.5 *μ*g/dL creates challenges as well as opportunities for state, local, and private laboratories that perform BLL testing. Some laboratories might need to reduce their reporting limit policies, adopt new repeat testing practices, improve limits of detection of laboratory developed tests, acquire new instrumentation, and validate updated or new laboratory-developed tests. Measures are also needed to eliminate lead contamination in laboratory consumables and processes and might increase workloads because of additional repeat and confirmatory testing. For example, skin prick tests can often be contaminated with environmental sources of lead, so collecting blood from the vein is less likely to have this contamination. Reducing the BLRV might strengthen considerations to tighten the federal proficiency testing criteria for acceptable blood lead testing performance from ±4 *μ*g/dL or ±10%, whichever is greater, to something tighter. Optimizing laboratory practices to meet the more stringent proficiency testing criteria might also be needed. Laboratory methods are sufficiently precise to measure BLLs at 3.5 *μ*g/dL.

HCPs play a vital role in addressing pediatric lead exposure by initiating recommended follow-up actions based on the child’s BLL. The updated BLRV empowers HCPs to take earlier action to mitigate exposures for children aged 1–5 years with BLLs between 3.5 and 5 *μ*g/dL, who, before this update, would not have been recommended to receive these services for a BLL <5 *μ*g/dL. Earlier recognition of lead exposure enables providers and families to intervene by stopping exposure that might otherwise result in higher BLLs, thus likely limiting or preventing potential adverse health effects.

Although there are practical challenges,** HCPs who identify children with BLLs between 3.5 and 5 *μ*g/dL should strive to ascertain possible sources of exposure by taking an environmental history and providing nutritional counseling which can help decrease lead absorption (*1*). Before this update, these actions were not recommended to occur for BLLs <5 *μ*g/dL. In addition, HCPs can provide guidance on exposure reduction, regardless of whether sources are identified, and link children to health departments for appropriate services. Follow-up testing using venous samples should be conducted after any remediation activities have taken place to ensure that exposure reduction was effective in addition to assessing developmental progress at regular intervals and providing referrals to supportive services as needed.

CDC’s CLPPP remains committed to eliminating lead hazards in the environment before children are exposed. Unfortunately, because lead hazards are ubiquitous in the environment, secondary prevention is necessary to identify and follow children who are exposed to lead. CDC recommends that public health and clinical professionals focus screening efforts on neighborhoods and children at high risk based on age of housing and sociodemographic risk factors. Public health and clinical professionals should collaborate to develop screening plans responsive to local conditions using local data. In the absence of such plans, universal BLL testing is recommended. In addition, jurisdictions should follow the Centers for Medicare & Medicaid Services requirement that all Medicaid-enrolled children be tested at ages 12 and 24 months or at age 24**–**72 months if they have not previously been screened (*3*).

Non-Hispanic Black children, those living in low-income households, and those who are immigrants or refugees are more likely to live in communities where lead is pervasive. These communities often have homes built before 1978, many of which contain lead-based paint. Although it is encouraging that progress has been made toward lowering population average BLLs, geographic, socioeconomic, and racial/ethnic disparities in lead exposure, especially among young children, persist. Efforts should be put in place focusing on eliminating lead exposure among the most vulnerable in the population, young children aged 1–5 years, and should become an environmental public health priority.

**Summary**

**What is already known about this topic?**

No safe blood lead level (BLL) in children exists. Even low levels cause harm.

**What is added by this report?**

CDC updated the blood lead reference value (BLRV) to 3.5 *μ*g/dL, which provides an opportunity for additional progress in addressing longstanding disparities in lead exposure and BLLs in children.

**What are the implications for public health practice?**

The BLRV should be used as a guide to empower public health partners to determine whether medical or environmental follow-up actions should be initiated for an individual child with BLLs between 3.5 and 5 *μ*g/dL who previously would not have been recommended to receive these services until their BLL reached 5 *μ*g/dL. In addition, it should be used to prioritize communities with the most need for primary prevention of exposure and evaluate the effectiveness of prevention efforts. Screening for BLLs should be done according to federal Medicaid and state requirements.

## Footnotes

* Water Infrastructure Improvements for the Nation Act of 2016. Pub. L. 114–322. https://www.congress.gov/bill/114th-congress/senate-bill/612/text

†
https://www.epa.gov/sites/default/files/2018-12/documents/fedactionplan_lead_final.pdf

§
https://cfpub.epa.gov/flpp/pub/index.cfm?do=main.firmSearch

¶
https://www.epa.gov/ground-water-and-drinking-water/lead-service-line-replacement

** According to an analysis from the Health Impact Project (https://www.pewtrusts.org/-/media/assets/2017/08/hip_childhood_lead_poisoning_report.pdf), eliminating lead hazards from the places where children live, learn, and play could generate approximately $84 billion in long-term benefits per birth cohort. In addition, permanently removing lead hazards from the environment would benefit future birth cohorts, and savings would continue to grow over time.
